# Superior triacylglycerol (TAG) accumulation in starchless mutants of *Scenedesmus obliquus*: (I) mutant generation and characterization

**DOI:** 10.1186/1754-6834-7-69

**Published:** 2014-05-12

**Authors:** Lenny de Jaeger, Ruben EM Verbeek, René B Draaisma, Dirk E Martens, Jan Springer, Gerrit Eggink, René H Wijffels

**Affiliations:** 1 Bioprocess Engineering and AlgaePARC, Wageningen University and Research Centre, PO Box 8129, 6700 EV Wageningen, The Netherlands; 2 Unilever Research and Development Vlaardingen, Olivier van Noortlaan 120, 3133 AT Vlaardingen, The Netherlands; 3 Food and Biobased Research and AlgaePARC, Wageningen University and Research Centre, PO Box 17, 6700 AA Wageningen, The Netherlands

**Keywords:** Starchless mutant, Starch, Triacylglycerol (TAG), Biofuel, *Scenedesmus obliquus*, *Acutodesmus obliquus*, Metabolism

## Abstract

**Background:**

Microalgae are a promising platform for producing neutral lipids, to be used in the application for biofuels or commodities in the feed and food industry. A very promising candidate is the oleaginous green microalga *Scenedesmus obliquus*, because it accumulates up to 45% w/w triacylglycerol (TAG) under nitrogen starvation. Under these conditions, starch is accumulated as well. Starch can amount up to 38% w/w under nitrogen starvation, which is a substantial part of the total carbon captured. When aiming for optimized TAG production, blocking the formation of starch could potentially increase carbon allocation towards TAG. In an attempt to increase TAG content, productivity and yield, starchless mutants of this high potential strain were generated using UV mutagenesis. Previous studies in *Chlamydomonas reinhardtii* have shown that blocking the starch synthesis yields higher TAG contents, although these TAG contents do not surpass those of oleaginous microalgae yet. So far no starchless mutants in oleaginous green microalgae have been isolated that result in higher TAG productivities.

**Results:**

Five starchless mutants have been isolated successfully from over 3,500 mutants. The effect of the mutation on biomass and total fatty acid (TFA) and TAG productivity under nitrogen-replete and nitrogen-depleted conditions was studied. All five starchless mutants showed a decreased or completely absent starch content. In parallel, an increased TAG accumulation rate was observed for the starchless mutants and no substantial decrease in biomass productivity was perceived. The most promising mutant showed an increase in TFA productivity of 41% at 4 days after nitrogen depletion, reached a TAG content of 49.4% (% of dry weight) and had no substantial change in biomass productivity compared to the wild type.

**Conclusions:**

The improved *S. obliquus* TAG production strains are the first starchless mutants in an oleaginous green microalga that show enhanced TAG content under photoautotrophic conditions. These results can pave the way towards a more feasible microalgae-driven TAG production platform.

## Background

With decreasing fossil fuel deposits and an increasing world population, the need for alternative renewable food and energy resources such as biofuels is stronger than ever. Several crops are considered for both edible oils and biofuels production such as rapeseed, jatropha, corn, soybean and palm oil [1,2]. However, for food and energy purposes, these crops need to be produced on a massive scale [3]. This will dramatically impact the available fertile agricultural land for food production [1]. Alternatively, microalgae are photoautotrophic microorganisms acknowledged for the production of bulk chemicals, biofuels, food, feed and nutraceuticals [3,4], and can be cultivated on saline and non-fertile grounds.

Several studies have been carried out on the production of triacylglycerol (TAG) from microalgae for the generation of biodiesel or edible oils [1,4,5]. Microalgae show a high potential as TAG producing organisms compared to other photosynthetic organisms [1,6]. The mechanism of TAG production in microalgae is not yet fully understood, but the generally accepted hypothesis is that when microalgae are exposed to unfavourable growth conditions, such as extreme pH, nutrient limitation or high salinity, they channel the excess energy from light into storage compounds such as starch and TAG [6-8]. These storage compounds serve as electron sinks, that alleviate an over-reduced photosystem, and in this way prevent the formation of reactive oxygen species, which can induce photooxidative stress [6,9].

The rate at which starch and TAG are accumulated *in vivo* under growth-limiting conditions is dependent on the microalgae species and cultivation conditions used [10]. *Chlamydomonas reinhardtii* is known for the production of large amounts of starch granules up to 45% of cell dry weight (DW) [11], whereas oleaginous microalgae such as *Scenedesmus obliquus* (recently suggested to be reclassified to *Acutodesmus obliquus*[12]) accumulate, besides starch, lipid bodies rich in TAG [10,13]. Generally, starch is accumulated from the onset of starvation and TAG accumulation is commenced a few hours to days later [14]. Although *S. obliquus* is referred to as an oleaginous species, other carbon containing compounds are produced as well. In the study of Breuer *et al*., the predicted carbohydrate content of *S. obliquus* is 40 to 60% of the dry weight [10] and TAG contents of 30 to 45% of the dry weight were observed [7]. Furthermore, the total fatty acid (TFA) composition is very suitable for the production of biodiesel due to the low content of linolenic and other polyunsaturated fatty acids, and the relative abundance of saturated and monounsaturated fatty acids [15].

**Figure 1:**
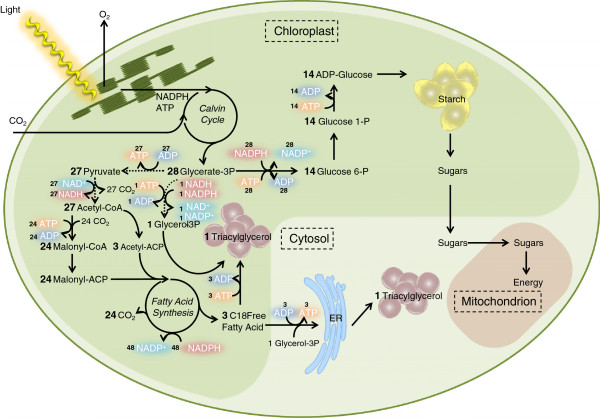
**Simplified triacylglycerol and starch metabolism in green microalgae.** Not all reactions are shown, and the cofactors upstream of 3PG are not considered. The dashed lines are reactions that take place in the cytosol. From 28 molecules of 3PG, one molecule of C18 TAG can be formed or 14 molecules of glucose in a starch matrix. Two possible ways for the formation of TAG molecules are shown following the postulated route in the chloroplasts or over the ER membranes in the cytosol [17-19]. 3PG, 3-phosphoglycerate; ER, endoplasmic reticulum; TAG, triacylglycerol.

Although there is no complete understanding of the mechanism behind carbon partitioning and the switch from starch towards TAG production, parts of the puzzle are known. Figure 1 shows a schematic overview of the carbon partitioning in microalgae. There is a common denominator in the carbon metabolism, the C3 pool, such as 3-phosphoglycerate (3PG) and glyceraldehyde 3-phosphate (GAP). The competition for those substrates is commonly referred to as carbon partitioning. Starch functions as a primary energy investment that can be stored during the day and used at night to provide energy for key metabolic processes [16]. TAG is considered as a secondary energy and electron sink [6], although not much is known about the regeneration of the energy stored in TAG molecules after a period of growth inhibition.

Since both TAG and starch compete for carbon, through the common C3 precursors, it was hypothesized that when the pathway towards starch formation is blocked, the carbon and energy flux towards TAG molecules is enhanced. This hypothesis was confirmed in starchless mutants (slm) of *C. reinhardtii*[20,21]. Li *et al.* compared TFA accumulation in wild type (wt) *C. reinhardtii* with five low starch or starchless mutants under nitrogen-limited conditions (I7, JV45J, BAFJ5, CR102, BAFJ6 [22-26]). They showed that the starch content of these mutants was lowered or even completely absent resulting in an increased TFA content compared to the wild type [11]. The starchless mutants showed that the TAG content could be increased from 0.5 to 20.5% (% of DW). Although the TAG content increased, growth kinetics of the starchless *C. reinhardtii* strains are severely subdued by the inserted mutation [20], resulting in decreased TAG productivity [11].

Besides the generation of starchless mutants using random mutagenesis, more directed approaches have been used as well. However, there are currently not many examples of successful enhancement of TAG content in microalgae using directed genetic engineering. Attempts to overcome bottlenecks in the TAG synthesis pathway, including overexpression of the enzymes acetyl-CoA carboxylase (ACCase) and the acyl-transferase step, 1,2-diacylgycerol acyltransferase (DGAT), did not yield enhanced TAG contents [27,28]. Recently Trentacoste *et al.*[29] showed that the knockdown of genes involved in the lipid catabolism can increase the TAG content in the diatom *Thalassiosira pseudonana*.

The fact that *C. reinhardtii* is not an oleaginous microalga by nature, but a starch producing microalga under unfavourable growth conditions, probably does not make this species the most suitable candidate to study lipid accumulation in starchless mutants. In this study, starchless mutants from the oleaginous microalga *S. obliquus* were generated to study the effect of blocked starch synthesis on TAG accumulation in a microalgae that by nature have the ability to accumulate high contents of TAG. Increasing the TAG productivity is an important step to enable a feasible microalgae-driven TAG production platform.

## Results and discussion

Previous studies on green microalgae starchless mutants have focussed mainly on the model species *C. reinhardtii*. The most promising starchless mutant known in *C. reinhardtii* is the BAFJ5 mutant containing a recessive mutation on the *sta6-1* gene encoding the small subunit of ADP-glucose pyrophosphorylase (AGPase) [26]. Under high light and nitrogen-limiting growth conditions, BAFJ5 accumulates up to 20.5% TAG (% of DW) under mixotrophic conditions (acetate) and around 14% under phototrophic conditions [11]. *C. reinhardtii* is not an oleaginous microalga by nature but blocking the starch metabolism induced the accumulation of TAG molecules under nitrogen-depleted conditions [11,20]. In this study we generated starchless mutants from the oleaginous green microalga *S. obliquus* and studied the effect on the TAG content of the mutants.

### ***S. obliquus*** mutant screening

**Figure 2:**
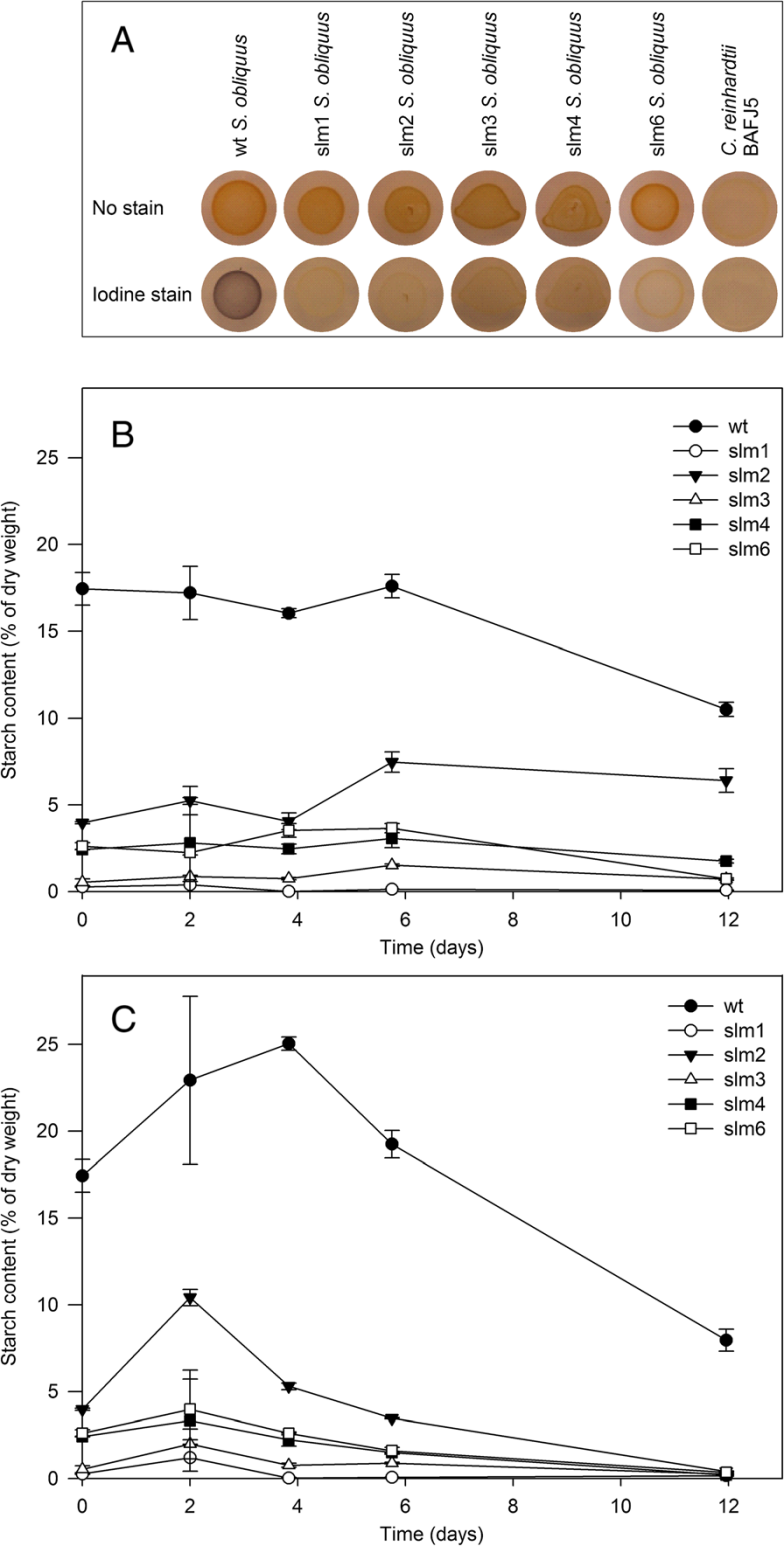
**Starch screening and content determination. (A)** Iodine vapour staining on starchless mutants. *C. reinhardtii* strain BAFJ5 was included as a control. The staining was performed after 9 days of nitrogen deprivation (TAP-N plates) to enhance the visibility of the iodine staining which is normally interfered by the chlorophyll. Starch content (% of DW) for **(B)** nitrogen-replete and **(C)** nitrogen-depleted conditions over time. The values are derived from biological duplicate cultures. Deviation from the duplicate average is indicated by the error bars. DW, dry weight; slm, starchless mutant; TAP, Tris-Acetate-Phosphate; wt, wild type.

Over 3,500 potential mutants of *S. obliquus* were obtained after UV irradiation, which were all screened for the absence of starch using the iodine vapour method. Wild type cells containing starch stained dark purple when exposed to iodine vapour (Figure 2A). Starchless mutants that stained pale green instead of purple were considered as potential starchless mutants and selected for further study. *C. reinhardtii* BAFJ5 was used as a positive control to check for reduced starch levels. In total, six colonies showed a reduced or absent colouring and were selected for further study (Figure 2A); slm5 was not included in this study because the growth under replete conditions was retarded in such a way that it could not be compared to the other strains. The five potential starchless *S. obliquus* mutants, as well as the wild type, were exposed to nitrogen starvation in a 100 mL shake flask cultivation experiment, to evaluate growth kinetics and biomass compositions. The biomass was examined on TFA, neutral lipid content (TAG) and starch content. The composition of the TFA and TAG was determined based on the fatty acid methyl esters (FAMEs).

### ***S. obliquus*** mutant biomass accumulation

**Figure 3:**
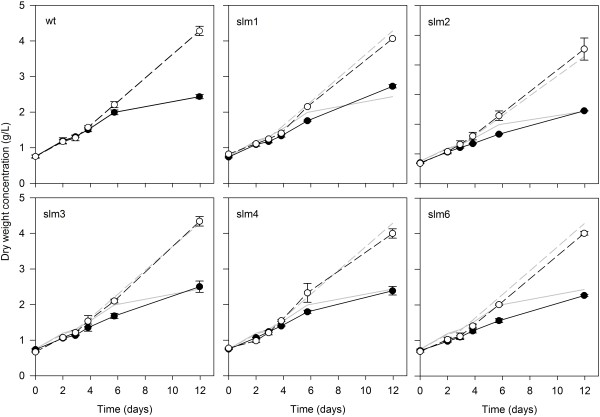
**Growth curves of *S. obliquus *wild type and five selected starch mutants.** Solid lines and filled circles represent strains cultivated under nitrogen-depleted conditions; dotted lines and open circles represent nutrient-replete conditions. The greyscale lines visualize the wild type growth curve in the starchless mutant plots. The values are derived from duplicate cultures. Deviation from the duplicate average is indicated by the error bars. slm, starchless mutant; wt, wild type.

Under nitrogen-replete conditions wild type *S. obliquus* accumulated biomass in a linear manner and reached around five times the start concentration after 12 days. Under nitrogen-depleted conditions the biomass increased around three times (Figure 3). This was also observed by Breuer *et al*. [10]. Interestingly, similar growth kinetics were observed in the five selected mutants, and all strains approached the final biomass concentration of the wild type (4.28 ± 0.04 g/L under nitrogen-replete conditions). Minor differences were observed in biomass concentration under nitrogen-replete conditions, and all mutants showed a slightly lower biomass concentration compared to the wild type. Under nitrogen-depleted conditions all *S. obliquus* potential starchless mutants had the same final biomass concentrations as the wild type (2.43 ± 0.04 g/L) except slm1 (2.72 ± 0.03 g/L), which was able to accumulate more biomass than the wild type.

Another way to study the growth kinetics is to examine the biomass productivity. In Table 1 the average and maximal volumetric biomass productivities (mg L^-1^ day^-1^) are shown. Under nitrogen-replete growth, slm2 had a higher average productivity compared to the wild type, with 322 ± 5 mg L^-1^ day^-1^ and 295 ± 2 mg L^-1^ day^-1^, respectively. Under nitrogen-depleted conditions, slm2, slm3 and slm4 exhibited a similar biomass productivity compared to the wild type (140 ± 5 mg L^-1^ day^-1^) ranging from 137 to 147 mg L^-1^ day^-1^. Slm6 had the lowest average biomass productivity, while slm1 showed the highest average biomass productivity, with 129 ± 1 mg L^-1^ day^-1^ and 166 ± 2 mg L^-1^ day^-1^, respectively (Table 1).

**Table 1:** **Volumetric productivities of *S. obliquus *** **wild type and five selected starchless mutants**

		**Volumetric biomass productivity (mg L**^**-1**^ **day**^**-1**^**)**		**Volumetric TFA productivity (mg L**^**-1**^ **day**^**-1**^**)**		**Volumetric TAG productivity (mg L**^**-1**^ **day**^**-1**^**)**
		**Average**	**Maximum**	**Average**	**Maximum**	**Average**
Wild type	N+	295 ± 2	334 ± 25	30 ± 2	42 ± 2	4 ± 0
	N-	140 ± 5	**255**^**b**^ **± 4**	104 ± 5	138 ± 5	95 ± 3
Slm 1	N+	271 ± 1	390 ± 24	35 ± 1	44 ± 2	3 ± 0
	N-	**166**^**b**^ **± 2**	222 ± 20	**125**^**b**^ **± 2**	**173**^**b**^ **± 9**	**112**^**b**^ **± 3**
Slm 2	N+	**322**^**a**^ **± 5**	362 ± 24	**42**^**a**^ **± 5**	54 ± 7	**12**^**a**^ **± 1**
	N-	146 ± 0	182 ± 22	101 ± 0	145 ± 2	94 ± 1
Slm 3	N+	307 ± 1	360 ± 9	42 ± 1	**57**^**a**^ **± 1**	3 ± 0
	N-	147 ± 1	240 ± 69	107 ± 1	170 ± 14	98 ± 2
Slm 4	N+	269 ± 2	**408**^**a**^ **± 87**	35 ± 2	46 ± 4	4 ± 0
	N-	137 ± 1	208 ± 24	105 ± 1	140 ± 6	95 ± 0
Slm 6	N+	278 ± 2	322 ± 8	35 ± 2	46 ± 4	7 ± 0
	N-	129 ± 1	187 ± 8	82 ± 1	115 ± 6	76 ± 1

Average productivities are calculated as the amount of TFA formed (mg) over the course of the experiment (12 days) per litre culture medium. Maximal productivities represent the highest productivity values between two consecutive time points. Average productivities are calculated for biomass, TFA and TAG. Maximal productivities are calculated for biomass and TFAs. Bold numbers represent the highest values for each parameter. ^a^Highest value for nitrogen-replete cultivated strains; ^b^highest value for nitrogen-depleted cultivated cultures. Average values of biological duplicate cultures are shown followed by the deviation of the duplicates from the average. slm, starchless mutant; TAG, triacylglycerol; TFA, total fatty acid.

Starch is an energetically low-cost storage metabolite, which through respiration functions as an energy source when photosynthesis is impaired. As starch is such an important storage component, its absence in starch mutants is expected to impair growth to a certain extent, as was observed in *C. reinhardtii*[11]. Li *et al.* showed a 30% growth reduction compared to the wild type under photoautotrophic conditions for the starchless mutant BAFJ5. The *S. obliquus* starchless mutants showed minor differences in the growth kinetics between the mutants and the wild type. This indicates that the metabolism of the mutants is not severely imbalanced and that *S. obliquus* found other ways to supply energy when photosynthesis is impaired, most likely through the use of the stored TAG.

### Biomass composition analysis

The oleaginous microalga *S. obliquus* is known for its ability to produce high amounts of TAG under suboptimal growth conditions [10,13]. Upon nitrogen starvation *S. obliquus* cells start to accumulate neutral lipid droplets inside the cell containing TAG molecules. Besides the accumulation of TAG molecules, starch is also accumulated [13]. It is hypothesized that mutants unable to accumulate starch will show an increased TAG content compared to the wild type, to counter the extra excess energy that cannot be stored in starch anymore upon growth inhibition. The major metabolite of interest for lipid production in microalgae is the TFA content. Even more so the TAG content that represents the neutral lipid fraction is of interest for the applications in food, feed and fuels.

### Starch

The wild type *S. obliquus* cells contained on average 17% of their dry weight as starch under nitrogen-replete growth in the first 6 days (Figure 2B). When cells were exposed to nitrogen-depleted conditions, the starch level increased to 25% of the dry weight within the first 4 days after medium replacement. Starch accumulation seemed to be a transient storage mechanism, since the starch content first increased and subsequently decreased to 10% after 12 days of nitrogen-depleted growth. All mutants showed a similar starch accumulation profile, but had a much lower starch content with respect to the wild type. Slm1 and slm3 showed the lowest starch content with a maximum of 1.2% and 2.0% of the dry weight, respectively (Figure 2C). These values are within the detection limit of the starch determination assay. This indicates that in these mutants the starch synthesis pathway was successfully impaired.

Several oleaginous microalgae are known to accumulate carbohydrates during the first days of nitrogen-depleted conditions [4,13,17]. The major form of carbohydrates that is accumulated is starch, and it is accepted that this metabolite functions as a primary carbon and electron storage compound. Starch serves as a primary storage compound, since the electrons required per unit of biomass are lower compared to TAG and proteins, which are more reduced than carbohydrates.

### Total fatty acid content and composition

**Figure 4:**
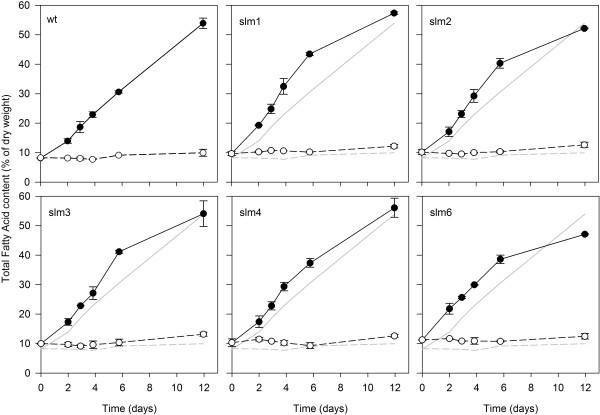
**Total fatty acid content of *S. obliquus *wild type and five selected starch mutants.** Values presented as percentage of dry weight. The dotted line and the solid line represent nitrogen-replete and nitrogen-depleted conditions, respectively. The greyscale lines in the plots represent the wild type TFA content. The values are derived from duplicate cultures. Deviation from the duplicate average is indicated by the error bars. slm, starchless mutant; TFA, total fatty acid; wt, wild type.

In Figure 4 it can be observed that under nitrogen-depleted conditions the TFA content is increased, and under nitrogen-replete growth no increase in the TFA content is observed. Under nitrogen-replete conditions all starchless mutants showed a higher TFA content compared to the wild type. An explanation could be that the absence of starch in the biomass makes the contribution of TFA compared to the dry weight slightly higher, therefore the relative TFA content would be higher. Another option could be that the internal TFA and TAG content was increased due to the blocked starch synthesis.

As a result of this impairment, the TFA content of all mutants increased after nitrogen starvation. All mutants showed an increased TFA accumulation rate during the first 6 days of nitrogen-depleted conditions. After 6 days the differences between the wild type and the mutants decreased, resulting in similar TFA contents after 12 days. Slm1 showed an increased TFA content (57.3 ± 0.4%) compared to the wild type (53.9 ± 1.8%) and slm6 seemed to lag behind (47.1 ± 0.3%). The TFA content of the starchless mutants showed a higher TFA accumulation rate after nitrogen starvation was induced, shortening the cultivation time in a batch culture.

**Figure 5:**
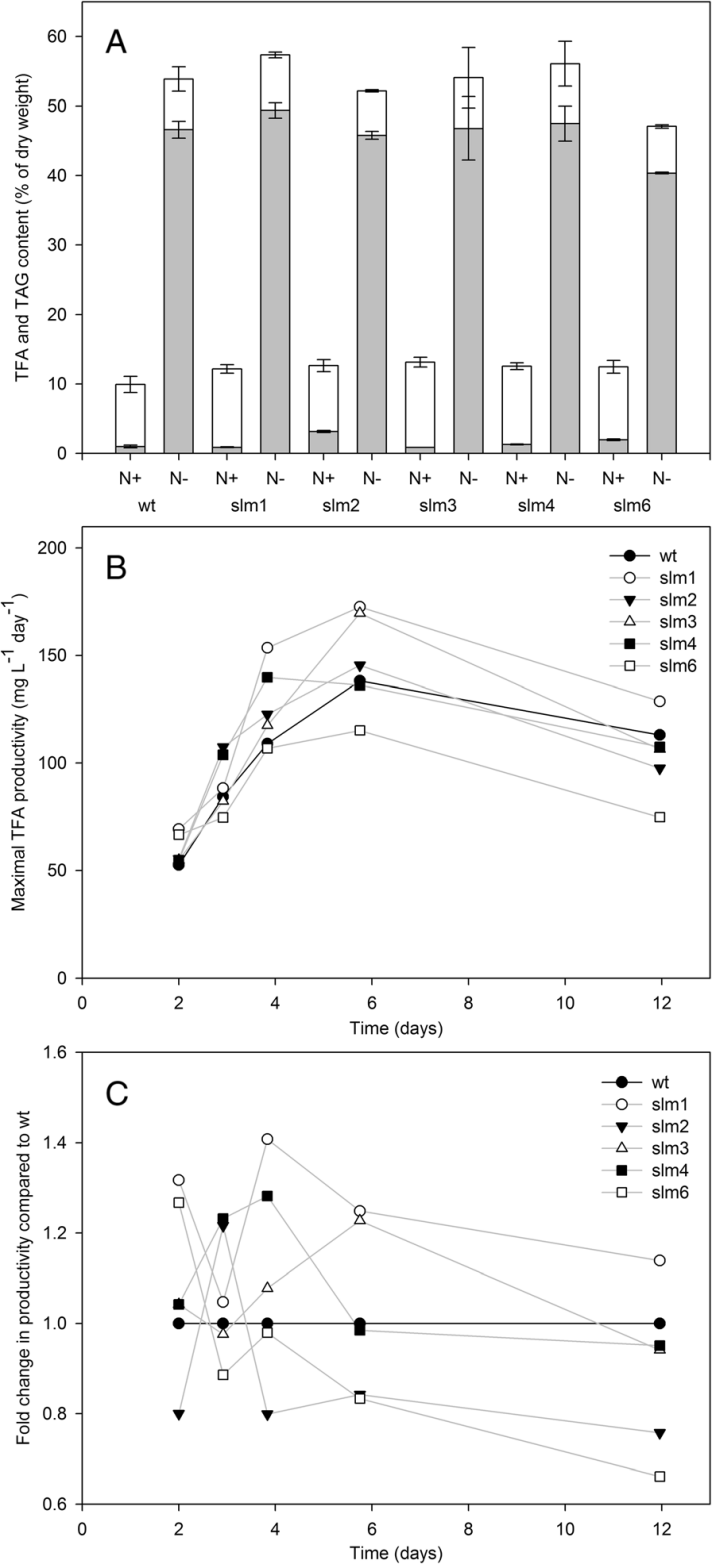
**Triacylglycerol content and total fatty acid productivity. (A)** Lipid content 12 days after nitrogen depletion. TAG (grey bars) and polar lipid (glycolipids and phospholipids) (white bars) content as percentage of dry weight. The values are derived from duplicate cultures. Deviation from the duplicate average is indicated by the error bars. **(B)** Productivity calculated between two consecutive time points in mg L^-1^ day^-1^. Wild type is represented by solid black line and the mutants are represented by grey lines. **(C)** Fold change in productivity (calculated between two consecutive time points) for all starchless mutants (greyscale) compared to the wild type (solid line). slm, starchless mutant; TAG, triacylglycerol; TFA, total fatty acid; wt, wild type.

The TFA content only partly shows the capacity of the selected starchless mutants to produce lipids. In order to assess the potential for lipid production of each strain, TFA productivity should be compared, since the TFA productivity represents the capacity of the strain to convert received light, which was the same for each culture, into the desired compound per unit of time. The differences in biomass concentration are taken into account, enabling a fair comparison of the TFA production by all strains tested. The initial biomass concentration at the start of nitrogen depletion was the same for all strains, thus an equal amount of light per gram of dry weight was supplied. The average productivity at which fatty acids were synthesized was higher under nitrogen-depleted conditions than under nitrogen-replete conditions (Table 1), with slm1 showing the highest average TFA productivity with respect to the wild type, 125 ± 2 mg L^-1^ day^-1^ compared to 104 ± 5 mg L^-1^ day^-1^, respectively; this corresponds to 20% more TFA than the wild type. Figure 5C shows that slm1 has a higher maximal TFA productivity compared to the wild type at every time point. The largest difference in maximal volumetric productivity is found between day 3 and day 4 after nitrogen starvation started, and slm1 accumulated 41% more TFA compared to the wild type (154 ± 40 mg L^-1^ day^-1^ and 109 ± 14 mg L^-1^ day^-1^, respectively) (Figure 5B,C). The second largest difference is at day 2, when slm1 accumulates 32.7% more TFA than the wild type (69 ± 4 mg L^-1^ day^-1^ and 52 ± 1 mg L^-1^ day^-1^, respectively). The maximum productivity, representing the productivity between two consecutive time points, was 25% higher in slm1 (173 ± 9 mg L^-1^ day^-1^) compared to the wild type (138 ± 5 mg L^-1^ day^-1^).

The TFA profiles of all strains were studied. The TFA and TAG fatty acid profiles are shown in Additional file 1: Table S1. There are no significant changes in the fatty acid profiles between the starchless mutants and wild type *S. obliquus*. This indicates that the fatty acid metabolism was not changed, and that the extra TFA and TAG formed in the mutants have the same composition and profile as wild type *S. obliquus*.

### Triacylglycerol

The TAG content was determined at the final time point, day 12 (Figure 5A). This was done because only here sufficient biomass was available for the analysis. Assuming that there is no polar lipid degradation or formation during nitrogen depletion, the TAG content in the intermediate time points can be calculated by subtracting the polar lipid fraction observed at the end of the nitrogen-replete cultivation, from the measured TFA fraction at each time point. Figure 5A shows that the difference in polar lipid fraction between nitrogen-replete and nitrogen-depleted cultures is small. Under nitrogen-replete growth the TAG content was low and ranged from 0.9 ± 0.0% to 3.2 ± 0.1% of total dry weight. The wild type contained around 1 ± 0.1% TAG (% of DW), which supports previous observations [10]. Slm6 and slm2 show a higher TAG content under nitrogen-replete growth conditions, with 2.0 ± 0.1% and 3.2 ± 0.1% (% of DW), respectively. This is in concurrence with our observation that throughout the entire nitrogen-replete experiment, starch mutants showed an elevated TFA content (Figure 4). *S. obliquus* wild type cells could accumulate 46.6 ± 0.9% of the cell dry weight in TAG, which represents 86% of the TFA content. Slm1 displayed a TAG content of 49.4 ± 0.8%, which represents 86% of the TFA content. Several studies have reported an inverse proportional relation between the lipid content and growth rate of the selected microalgae species. In other words, the higher the growth rate the lower the lipid content (% of DW), and vice versa [6,30,31].

In this study we show that the oleaginous microalga *S. obliquus* is able to accumulate more neutral lipids when the starch synthesis is blocked without considerably changing the biomass productivity. These observations suggest that *S. obliquus* produces TAG directly from light and CO_2_ and not from starch, and that the efficiency of light conversion to energy is not compromised. This is in contrast to other oleaginous microalgae. In the study of Li *et al. Pseudochlorococcum* sp. transient starchless mutants were studied [32]. The reduced starch content resulted in the reduction of TAG content, suggesting that in this species, starch functions as a feedstock for the subsequent production of TAG molecules. The same mechanism has been observed in higher plants [33]. The results from this study show that when starch synthesis is blocked the TAG content and productivity increases. It is important to realize that the TAG accumulation mechanism is not the same in all oleaginous microalgae. The differences in phenotype and metabolism between species show the importance of carefully selecting a species with the right traits specific for the product of interest.

### Industrial applications

In microalgae, starch can function as a primary carbon storage compound that is accumulated during the day and consumed during the night [16,34]. A similar phenomena is observed in the higher plant *Arabidopsis thaliana* in which starch respiration is under circadian control to ensure a sufficient carbohydrate supply until the next day [35]. Starchless mutants of *A. thaliana* are found to have a similar behaviour compared to the wild type when exposed to very long days or continuous light, but have an impaired growth when exposed to normal diurnal light cycles [36,37]. The starchless mutants of *S. obliquus* might therefore face problems to survive light-dark cycles. We therefore evaluated the growth of starchless mutants of *S. obliquus* under day-night cycles and found that this is not the case and all starchless mutants were able to grow in day-night cycles under replete conditions, although some starchless mutants showed a slightly altered growth rate (Additional file 2: Figure S1). It is very likely that another storage compound, likely TAG, is used as an energy and carbon source during the night.

The mutants generated by UV mutagenesis in this study need to be screened regularly for the stability of the mutation over multiple generations, as they do not contain a selection marker or any other trait to apply an external selection pressure to maintain the mutation. This is in contrast to transformants that are obtained by targeted transformation approaches or mutants obtained by insertion mutagenesis. A major advantage of UV-generated mutants is that for the large-scale outdoor cultivation and final use in food products no additional requirements and regulations are anticipated. The mutants presented in this work have shown to be stable over the course of 20 months. In particular, slm1, with its high TAG production rate and non-impaired growth kinetics, compared to the wild type under both continuous as well as light-dark illumination, would be a suitable candidate for large-scale, outdoor TAG production.

## Conclusion

Five starchless mutants of the oleaginous microalga *S. obliquus* were obtained by UV mutagenesis. Five starchless mutants were selected and showed a decreased or completely absent starch content. In parallel, an increased TAG accumulation rate was observed for the starchless mutants and no substantial decrease in biomass productivity was perceived. All mutants showed an increased TFA and neutral lipid productivity. Slm1 has a 41% higher TFA productivity compared to the wild type under phototrophic nitrogen-limiting conditions after 4 days of nitrogen depletion and reached a TAG content of 49.4% (% of DW). The increase in TFA is achieved without compromising the growth rates of the starchless mutants. Therefore, slm1 should be considered as a potential production strain for TAG, since the increased lipid productivity shortens the batch runtime with respect to the wild type, bringing economical microalgae biofuel production one step closer.

## Materials and methods

### Strain and mutant generation

The *S. obliquus* wild type strain (UTEX 393) (recently suggested to be reclassified to *A. obliquus*[12]) was acquired from the Culture Collection of Algae at the University of Texas at Austin (UTEX; Austin, TX, USA). The culture was maintained on Tris-Acetate-Phosphate (TAP) medium as described by Gorman and Levine [38], with addition of 15 g/L agar for plates.

Approximately 20,000 cells in late log phase (1 × 10^7^ cells/mL) were plated on TAP agar plates and air-dried for 30 min before UV treatment. Subsequently, the cultures were exposed to 40,000 μJ cm^-1^ of monochromatic UV light with a wavelength of 254 nm (CL-1000 UV crosslinker; UVP, Upland, CA, USA) at room temperature, resulting in a 5 to 10% survival rate. The total dose of radiation was determined by the UV sensor equipped with the instrument.

After irradiation, plates were stored overnight in the dark to reduce light-induced repair mechanisms. Subsequently, plates were incubated in 50 μmol m^-2^ s^-1^ light under continuous illumination at 25°C for 10 days, after which single colonies were picked and transferred to fresh TAP plates and categorized in a grid. As a positive control, every plate contained a patch of the *C. reinhardtii* starch mutant BAFJ5 [26].

Plates were cultivated under the same conditions for 5 days, after which all colonies were replica plated to TAP-N (NH_4_Cl was omitted and substituted by an equimolar amount of KCl) plates in the same grid. After 9 days of incubation the colonies were screened for the presence of starch by applying an iodine vapour staining [39]. Colonies that did not stain purple after the iodine vapour treatment were considered as potential starchless mutants, and selected for further analysis.

### Triacylglycerol induction in starchless mutants of ***S. obliquus***

Pre-cultures were maintained photoautotrophically under day-night regime (16 h:8 h) at 25°C, 125 rpm and under an incident light intensity of 40 μmol m^-2^ s^-1^. The wild type and all starchless mutants were able to cope with multiple serial dilutions. The photoautotrophic culture medium that was used during the experiments comprised of: 33.6 mM KNO_3_ (in nitrogen-replete medium, N+) or 33.6 mM KCl (in nitrogen-depleted medium, N-); 0.7 mM Na_2_SO_4_; 100 mM 2-(4-(2-hydroxyethyl)piperazin-1-yl)ethanesulfonic acid (HEPES); 1 mM MgSO_4_ · 7H_2_O; 0.5 mM CaCl_2_ · 2H_2_O; 2.5 mM K_2_HPO_4_; 10 mM NaHCO_3_; 28 μM NaFeEDTA; 80 μM Na_2_EDTA · 2H_2_O; 19 μM MnCl_2_ · 4H_2_O; 4 μM ZnSO_4_ · 7H_2_O; 1.2 μM CoCl_2_ · 6H_2_O; 1.3 μM CuSO_4_ · 5H_2_O; and 0.1 μM Na_2_MoO_4_ · 2H_2_O. The pH was adjusted to pH 7.0 with NaOH.

To study the growth of the wild type and all slm, two cultivation experiments were conducted in which all strains (triplicates) were exposed to nitrogen-replete photoautotrophic conditions under 16 h:8 h day-night regime for 5 to 7 days in a 6-well plate containing 5 mL culture volume. Growth was followed by OD_750_ measurements using the Tecan Safire plate reader (Tecan Austria GmbH, Grödig, Austria).

To evaluate the TAG producing capacity of the selected mutants, cultures were maintained in 115 mL photoautotrophic medium in 250 mL glass Erlenmeyer flasks under an incident light intensity of 60 μmol m^-2^ s^-1^ (continuous illumination, Grolux fluorescent tubes, Sylvania F36W/GRO) at 25°C in an Multitron incubator shaker (Infors AG, Bottmingen, Switzerland). Agitation was kept at 125 rpm and the headspace was enriched to 5% CO_2_.

Two flasks of wild type *S. obliquus*, as well as all selected mutants, were grown under nitrogen-replete conditions to a biomass concentration of 1.5 g/L. Duplicate flasks were pooled to provide sufficient starter material, and divided into four aliquots with an equal cell concentration. The aliquots were washed (centrifuged for 5 minutes at 400 × *g*) two times with either medium containing 33.6 mM KNO_3_ (N+) or medium with an equimolar amount of KCl (N-), and subsequently resuspended in either nitrogen-replete or nitrogen-depleted medium. This step is considered as T0. As such, duplicate cultures were available for all tested mutants and the wild type, under both N- and N + conditions.

Culture development was studied over a period of 12 days. Samples were taken at 0, 48, 70, 92, 138 and 285 hours, to evaluate optical density, dry weight and biomass composition. For every time point, TFA and starch contents were analyzed, and for the last time point the TAG content was determined. For all parameters biological replicates were examined.

### Determination of dry weight concentration

Dry weight concentrations were determined at every time point on biological replicates. Around 1.5 mg of biomass was filtered through pre-dried (100°C overnight) and pre-weight Whatman glass fibre filter paper (GF/F; Whatman International Ltd, Maidstone, UK). The filter was washed with 50 mL of filtered demineralized water and subsequently dried overnight at 100°C before weighing.

### Total fatty acid analysis

TFA extraction and quantification were executed as described by Breuer *et al*. [40] with the following adjustments. Around 5 mg of pellet was transferred to bead beating tubes (Lysing Matrix E; MP Biomedicals, Santa Ana, CA, USA) and lyophilized overnight. Freeze-dried cells were disrupted by a 30 min bead beating step in the presence of a chloroform:methanol mixture (1:1.25) to extract the lipids from the biomass. Next, 48.6 μg/mL tripentadecanoin (T4257; Sigma-Aldrich, St Louis, MO, USA) internal standard was added to the extraction mixture to enable fatty acid quantification. Methylation of the fatty acids to FAMEs and the quantification of the FAMEs were performed as described by Breuer *et al.*[40]. TFA concentration was calculated as the sum of all individual fatty acids.

### Triacylglycerol analysis

The method used for the quantification of TAG was similar to the TFA analysis method with the following modifications. After the TFA extraction, the chloroform methanol mixture was evaporated under N_2_ gas and the TFA fraction was dissolved in 1 mL hexane and separated based on the polarity using a Sep-Pak Vac silica cartridge (6 cc, 1,000 mg; Waters, Milford, MA, USA) prewashed with 6 mL of hexane. The neutral TAG fraction was eluted with 10 mL of hexane-diethyl ether (87:13% v/v). The polar lipid fraction containing the glycolipids and phospholipids remained in the silica cartridge. Subsequently, the TAG fraction was methylated and analyzed as described in the TFA analysis section.

### Starch determination

The starch content of *S. obliquus* was analyzed by enzymatic degradation of starch to glucose using the thermostable α-amylase and amyloglucosidase enzymes from the Total Starch assay (Megazyme International, Wicklow, Ireland) using the protocol as described by de Winter *et al.*[16]. The following modifications to the protocol were made. Around 10 mg of biomass was transferred to bead beating tubes (Lysing Matrix E; MP Biomedicals) and lyophilized overnight. Freeze-dried cells were disrupted by bead beating in the presence of 80% ethanol. Starch was converted to glucose using α-amylase and amyloglucosidase enzymes. Subsequently glucose was coloured and absorbance was measured against a D-glucose calibration control series at a wavelength of 510 nm.

## Abbreviations

3PG: 3-Phosphoglycerate; ACCase: Acetyl-CoA carboxylase; AGPase: ADP-glucose pyrophosphorylase; DGAT: Diacylgycerol acyltransferase; DW: Dry weight; EDTA: Ethylenediaminetetraacetic acid; FAME: Fatty acid methyl ester; GAP: Glyceraldehyde 3-phosphate; HEPES: 2-(4-(2-Hydroxyethyl)piperazin-1-yl)ethanesulfonic acid; OD: Optical density; slm: Starchless mutant; TAG: Triacylglycerol; TAP: Tris-Acetate-Phosphate; TFA: Total fatty acid; wt: Wild type.

## Competing interests

RD is employed by Unilever; however, this does not alter the authors’ adherence to the *Biotechnology for Biofuels* policies on sharing data and materials. This study has been carried out in research and development collaboration between Wageningen UR and Unilever Research and Development Vlaardingen. In general, Unilever is interested in the potential of microalgae as an alternative sustainable source of oils. All other authors declare that they have no competing interests.

## Authors’ contributions

LdJ carried out the design of the experiments, the performance of the experiments and wrote the manuscript. RV carried out the growth experiment and assisted with the manuscript. RD edited the manuscript. DM, JS, GE and RW conceived of the study, participated in its design and coordination, and edited the manuscript. All authors read and approved the final manuscript.

## Supplementary Material

Additional file 1: Table S1 Fatty acid composition expressed as percentage of TFAs or fatty acids in TAG 12 days after medium replacement. slm, starchless mutant; TAG, triacylglycerol; TFA, total fatty acid; wt, wild type. Click here for file

Additional file 2: Figure S1 Growth curve for starchless mutants and wild type under day-night regime. OD, optical density; slm, starchless mutant. Click here for file
