# Supplementary material for: Superior triacylglycerol (TAG) accumulation in starchless mutants of Scenedesmus obliquus: (I) mutant generation and characterization
Source: Biotechnol Biofuels. 2014 May 12;7:69. doi: 10.1186/1754-6834-7-69 (PMC4052810; doi:10.1186/1754-6834-7-69)
Supplement: Additional file 1: Table S1 — Fatty acid composition expressed as percentage of TFAs or fatty acids in TAG 12 days after medium replacement. slm, starchless mutant; TAG, triacylglycerol; TFA, total fatty acid; wt, wild type. [file 1754-6834-7-69-S1.pdf]

Supplementary data 1

Table 1 Fatty acid composition expressed as % of total fatty acids (TFA) or fatty acids in TAG (TAG) 12 days after medium replacement. (A) Average fatty acid composition of TFA and TAG during nitrogen replete cultivation. (B) Fatty acid composition of TFA and TAG under nitrogen deplete conditions. Numbers given are averaged values (n=2) and the deviation from the duplicate average was less than 1% for all values except for the values that are indicated by an asterix, where the deviation was between 1% and 5%.

(A)	wild type		slm1		slm2		slm3		slm4		slm6	
	TFA	TAG	TFA	TAG	TFA	TAG	TFA	TAG	TFA	TAG	TFA	TAG
C16:0	16.2	11.2	16.0	11.2	16.2	12.5	16.3	13.1	16.6	15.4	14.9	10.6
C16:1	2.6	1.7	2.6	0.1	2.3	1.9	2.6	0.6*	2.5	2.0	2.3	1.8
C16:2	2.4	1.1	3.0	1.3	1.9	0.9	2.6	1.2	2.5	0.5	2.3	1.1
C16:3	3.4	1.4	4.4	1.7	5.5	1.5	3.6	1.2	4.1	1.4	3.6	0.7
C16:4	11.1	3.6	10.5	3.5	7.8	2.7	11.5	3.7	10.9	2.7	10.7	3.9
C18:0	0.6	2.2	0.5	2.2	0.7	1.3	0.6	3.0	0.8	2.2	0.6	1.5
C18:1	14.8	43.6	12.3	43.1	24.5	54.2	12.2	42.1	14.3	46.6*	17.1	47.0
C18:2	19.9	11.5	23.1	13.2	13.0	6.0	21.1	11.5	18.2	8.1	17.6	10.5
C18:3	18.7	10.2	18.7	11.1	10.3	11.6	18.9	11.6	19.8	11.1	19.6	11.1
C18:4	5.1	6.6	4.7	5.9	5.6	5.8	5.2	5.8	5.5	5.4	5.9	6.8
Other	5.2	6.8	4.2	7.5	12.2	1.5	5.4	6.2	5.0	4.5	5.6	5.0
(B)												
C16:0	16.5	16.2	16.4	16.2	18.0	18.0	17.8	17.7	17.9	17.8	15.8	15.4
C16:1	3.8	4.0	4.4	4.6	4.1	4.3	3.9	4.1	3.6	3.8	3.7	3.9
C16:2	2.8	2.6	2.9	2.7	2.4	2.3	2.6	2.5	2.0	2.0	2.8	2.7
C16:3	2.4	2.0	2.7	2.3	2.5	2.2	2.5	2.2	2.7	2.3	2.7	2.3
C16:4	2.0	1.6	1.5	1.2	1.7	1.5	1.8	1.5	1.9	1.5	2.4	1.9
C18:0	3.6	3.9	3.8	4.1	4.1	4.3	4.1	4.3	3.9	4.1	3.1	3.3
C18:1	47.9	51.2	49.5	52.4	47.9	50.4	47.4*	50.1	48.1*	50.9	47.7	50.6
C18:2	8.2	7.7	7.0	6.5	7.0	6.6	7.6	7.1	6.6	6.3	7.9	7.6
C18:3	10.3	8.7	9.6	8.3	10.1	8.7	10.0	8.7	10.8	9.4	11.7	9.9
C18:4	1.2	1.1	1.0	0.9	1.1	1.0	1.1	1.0	1.3	1.2	1.4	1.2
Other	1.2	0.9	1.0	0.8	1.0	0.8	1.2	0.8	1.3	0.8	0.8	1.0
