# Supplementary material for: Superior triacylglycerol (TAG) accumulation in starchless mutants of Scenedesmus obliquus: (I) mutant generation and characterization
Source: Biotechnol Biofuels. 2014 May 12;7:69. doi: 10.1186/1754-6834-7-69 (PMC4052810; doi:10.1186/1754-6834-7-69)
Supplement: Additional file 2: Figure S1 — Growth curve for starchless mutants and wild type under day-night regime. OD, optical density; slm, starchless mutant. [file 1754-6834-7-69-S2.pdf]

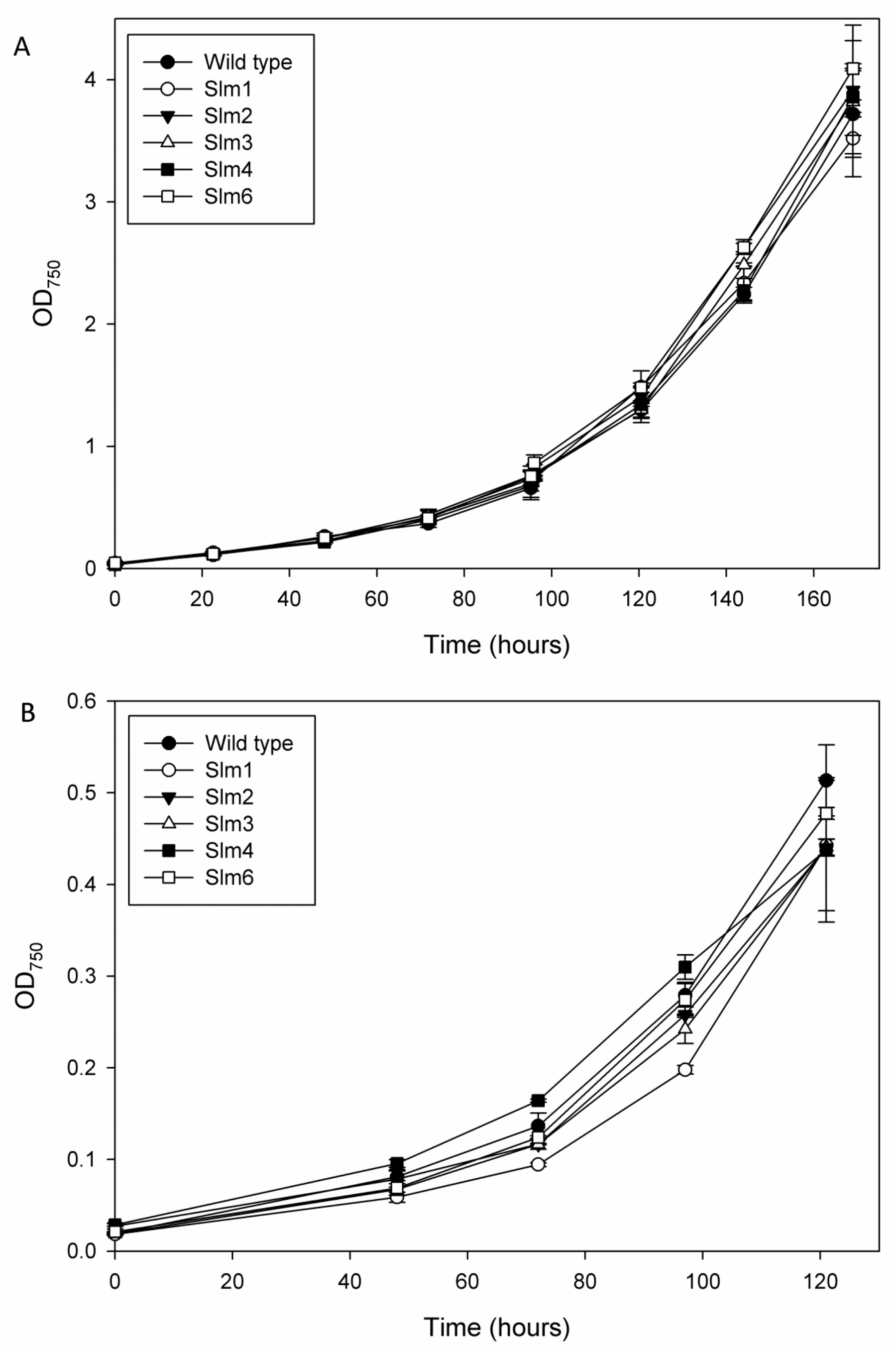

Growth curve for starchless mutants and wild type under day night regime. Two separate experiments were carried out to study the effect of day night cycles on all strains. 16:8 hour day night regime was applied. The growth was studied by measuring the OD_{750} daily. (A) Cultivation in triplicate cultures for 7 days. (B) Cultivation in duplicate cultures for 5 days. Error bars indicate the distance to the mean. It can be seen that there are not many differences between the wild type and starchless mutants under these conditions.
